# Unmasking the Emotional and Behavioral Determinants of Glycemic Control in Diabetes Management

**DOI:** 10.7759/cureus.92635

**Published:** 2025-09-18

**Authors:** Shouvik Ganguly, Ipseeta Ray Mohanty, Sandeep Rai

**Affiliations:** 1 Pharmacology, MGM Medical College, Navi Mumbai, Navi Mumbai, IND; 2 Internal Medicine, MGM Medical College, Navi Mumbai, Navi Mumbai, IND

**Keywords:** diabetic distress, glycemic control, medication adherence, self-care management, type 2 diabetes mellitus

## Abstract

Background

In type 2 diabetes mellitus (T2DM), glycemic regulation is influenced not only by physiological parameters but also by behavioral patterns, emotional states, and socioeconomic variables. These nonclinical factors are increasingly recognized for their potential role in personalizing diabetes care.

Objectives

This study examined the influence of behavioral factors (such as medication adherence and diabetes self-care), diabetes-related emotional distress, and socioeconomic status, in addition to clinical determinants, on glycemic control among urban Indian individuals with T2DM.

Methods

This cross-sectional investigation enrolled adult patients with T2DM. Socioeconomic classification was performed using the Modified Kuppuswamy Scale (2022). Medication adherence was assessed with the Morisky Medication Adherence Scale 4-item, while behavioral and emotional components were evaluated using the Diabetes Self-Management Questionnaire and the Diabetes Distress Scale. Comprehensive patient profiles, including anthropometry, lipid panels, and body-fat metrics, were collected. Associations between the variables of interest and glycemic status were examined using chi-square analysis.

Results

Higher self-management scores were significantly associated with favorable glycemic profiles (χ² = 9.574, p = 0.008). Elevated diabetes-related emotional distress showed a strong association with glycemic outcomes (χ² = 9.6824, p = 0.007), even among patients who maintained their glycemic targets. Notably, all subjects reported some degree of distress. Socioeconomic factors, central obesity, and triglyceride and high-density lipoprotein levels influenced adherence patterns and access to care.

Conclusions

Effective glycemic control in T2DM is closely linked to behavioral adherence and emotional resilience. These findings underscore the importance of integrative, patient-tailored approaches that account for psychological and contextual factors, particularly in culturally diverse and resource-variable settings.

## Introduction

Type 2 diabetes mellitus (T2DM) is a progressive metabolic disorder characterized by insulin resistance and impaired pancreatic β-cell function, resulting in chronic hyperglycemia. Effective glycemic control, usually assessed by glycated hemoglobin (HbA1c), is essential for reducing the risk of both microvascular and macrovascular complications, including retinopathy, nephropathy, neuropathy, and cardiovascular disease [1]. Despite the availability of pharmacologic therapies and structured diabetes education programs, many patients fail to achieve target glycemic levels [2].

Maintaining stable blood glucose levels is a cornerstone of diabetes care, as it minimizes both immediate and long-term health risks [3]. Poorly controlled diabetes can result in severe complications affecting the eyes, kidneys, nerves, and cardiovascular system. Landmark studies such as the UK Prospective Diabetes Study demonstrated that even a 1% reduction in HbA1c significantly reduces risks: diabetes-related mortality by 21% and microvascular complications by 37% [4,5]. These findings underscore the importance of early detection, individualized treatment strategies, and consistent monitoring to improve glycemic outcomes and reduce the burden of complications.

Glycemic regulation is influenced by a complex interplay of factors. Patient-specific variables such as age, sex, BMI, central adiposity, socioeconomic status, and psychological distress are strongly associated with glycemic outcomes [6]. Behavioral determinants, including adherence to self-care practices such as glucose monitoring, dietary compliance, physical activity, and healthcare engagement, also play a pivotal role in daily management. In addition, disease-related factors such as age at onset, duration of diabetes, family history, and comorbidities (e.g., hypertension or dyslipidemia) further influence glycemic stability [2]. Therapeutic determinants, including treatment modality (monotherapy versus combination therapy), pharmacological class, and adherence, directly affect the success of glycemic control [7].

Recent evidence shows that younger age (<60 years), longer disease duration (>10 years), limited diabetes knowledge, and poor self-care behaviors are strongly associated with suboptimal glycemic control [7,8]. Socioeconomic disparities and restricted access to healthcare exacerbate these challenges, particularly in resource-constrained settings. Early identification of these factors and targeted interventions are crucial for improving glycemic outcomes and reducing the overall disease burden [9].

Although glycemic control in T2DM has been extensively studied, most research has focused on sociodemographic, anthropometric, clinical, behavioral, or psychosocial variables in isolation, leading to fragmented insights that limit the development of integrated, patient-centered interventions [2,7]. This gap is especially pronounced in underrepresented populations, where multidimensional and context-sensitive approaches are required. Furthermore, standardized tools such as the Morisky Medication Adherence Scale 4-item (MMAS-4), the Diabetes Self-Management Questionnaire (DSMQ), and the Diabetes Distress Scale (DDS) are rarely applied together.

This study addresses these limitations by integrating all three scales to examine the interplay of medication adherence, self-care behaviors, emotional distress, and socioeconomic factors with glycemic outcomes across diverse socioeconomic strata in urban India. We hypothesized that, independent of standard clinical parameters, nonclinical factors, specifically medication adherence, diabetes-related distress, self-care behaviors, and socioeconomic status, would show significant associations with glycemic control. The aim of this study is therefore to provide a multidimensional perspective on glycemic regulation in T2DM.

## Materials and methods

Ethics approval declaration

The study was approved by the Ethics Committee for Research on Human Subjects, Re-Registration No. ECR/457/Inst/MH/2013/RR-20, MGMIHS, Navi Mumbai, India (approval MGMIHS: Res.:02:2022:646).

Study design and data collection

This was a cross-sectional clinical study conducted among patients with T2DM. The study period extended from August 1, 2023, to January 31, 2025.

Study Population

A total of 310 T2DM patients who met the specified inclusion criteria were included in the study.

Inclusion Criteria

Adults aged 18 to 65 years, diagnosed with T2DM based on the American Diabetes Association criteria, and undergoing treatment for at least four months, were eligible for inclusion. Patients with comorbidities were also included. Written informed consent was obtained from all participants prior to enrollment.

Exclusion Criteria

Patients with type 1 diabetes mellitus, pregnant women (including those with gestational diabetes), patients with diabetes secondary to other factors (e.g., malnutrition, infection, and surgery), patients unable to complete the questionnaire, and those who were hospitalized and/or had a psychiatric disorder during the data collection period were excluded.

Sample size

The sample size was calculated based on the study by Borgharkar and Das [10], which reported 76.6% of T2DM patients having poor glycemic control. To account for potential nonresponses and missing data, 10% was added to the calculated sample size: n ≈ 282 × 1.1 (10% increase). Therefore, 310 patients were included in the study.

Case record form (CRF)

A CRF was prepared to collect information from T2DM patients attending the medicine outpatient department. Glycemic status was assessed using HbA1c levels, with patients categorized as having good glycemic control (HbA1c <7.0%) or poor glycemic control (HbA1c ≥7.0%). In addition to HbA1c, fasting and postprandial blood glucose values were recorded to support glycemic profiling. Sociodemographic data, including age, gender, occupation, educational level, and family income, were obtained for each participant. Based on these variables, individuals were classified into five socioeconomic strata (upper, upper middle, lower middle, upper lower, and lower) according to the modified 2022 version of Kuppuswamy’s Socioeconomic Status Scale, which is freely available for academic and noncommercial research purposes [11].

Patients’ anthropometric and metabolic profiles included BMI, waist-to-hip ratio (WHR), lipid profile, and body fat analysis results. A BMI of <25 was considered normal, 25-29.5 overweight, and >30 kg/m² obese. Cardiometabolic risk was assessed using both anthropometric and biochemical parameters. WHR was considered elevated if it exceeded 0.90 in males or 0.85 in females. Lipid profile evaluation included total cholesterol, high-density lipoprotein (HDL), low-density lipoprotein (LDL), and triglycerides (TG), with total cholesterol <200 mg/dL classified as normal, HDL <40 mg/dL in males and <50 mg/dL in females considered low, LDL >100 mg/dL abnormal, and TG >150 mg/dL abnormal. Body fat analysis was also performed to measure body fat percentage, resting metabolic rate, and visceral fat level [12].

The disease profile included age at onset of T2DM, family history of diabetes, smoking status, blood pressure (>130/80 mmHg or use of antihypertensive drugs), and abnormal lipid levels. Treatment modalities prescribed to each patient were also recorded.

Medication adherence was assessed using the MMAS-4 developed by Morisky et al. [13]. The scale is copyrighted, and permission was obtained from the developer, Prof. Donald E. Morisky (certificate number: 1390695790). The four questions included in the MMAS-4 are: “Do you ever forget to take your medicine?”, “Are you ever careless about taking your medicine?”, “When you feel better, do you sometimes stop taking your medicine?”, and “Sometimes you feel worse when you take the medicine; do you stop taking it?”

Diabetes self-care was assessed using the DSMQ, which is freely available for academic and noncommercial research purposes. The questionnaire provides a total score (“sum scale”) and four subscale scores: Glucose Management, Dietary Control, Physical Activity, and Health Care Use [14].

Diabetes distress was assessed using the DDS total score, which reflects the patient’s overall diabetes distress. The DDS is also freely available for academic and noncommercial research purposes [15].

Study procedure

All diabetic patients attending the medicine outpatient department who met the inclusion criteria were enrolled after the aim of the study was explained. Written informed consent was obtained from each participant. Approval from the institutional ethics committee, the hospital superintendent, and the head of the medicine department was secured prior to the study. Patients who had been receiving any antidiabetic drug or insulin (or both) for at least four months were included, irrespective of gender. Blood pressure and anthropometric parameters were measured, and other details specified in the CRF were collected. Blood samples were analyzed in the central laboratory for HbA1c and lipid profile levels. Based on HbA1c values, patients were divided into “good” and “poor” glycemic control groups. Data were collected and analyzed to identify factors associated with glycemic control.

Data analysis and statistical methods

Descriptive statistics were used to summarize patient demographics and survey responses. Differences in HbA1c outcomes were evaluated using t-tests for continuous variables and chi-square tests for categorical variables. P-values <0.05 were considered statistically significant. Statistical analyses were conducted using IBM SPSS Statistics for Windows, Version 25.0 (Released 2017; IBM Corp., Armonk, NY, USA).

## Results

Glycemic control status

Among the 310 sampled patients, 83 individuals (26.8%) had good glycemic control (HbA1c < 7.0), whereas 227 (73.2%) exhibited poor control (HbA1c ≥7.0).

Sociodemographic profile

Socioeconomic Status

A statistically significant association was observed between socioeconomic status and glycemic control (χ² = 10.533, p = 0.015). Most patients with good glycemic control (75.9%) belonged to the upper lower class (Class IV), followed by 18.1% from the lower class (Class V). The majority of patients with poor glycemic control (63.9%) belonged to the upper lower class, followed by 21.1% from the lower class. These results indicate that socioeconomic status significantly impacts glycemic control (Table 1).

**Table 1:** Sociodemographic factors affecting glycemic control

Variables	Good glycemic control		Poor glycemic control		Total	Chi-square test	p-Value	Significant at the 5% level
	f	%	f	%				
Age group (years)								
18-35	0	0	14	6.2	14	6.179	0.103	No
36-45	22	26.5	64	28.2	86			
46-60	58	69.9	138	60.8	196			
60-65	3	3.6	11	4.8	14			
Total	83	100	227	100	310			
Gender								
Female	44	53	113	49.8	157	0.254	0.614	No
Male	39	47	114	50.2	153			
Socioeconomic class								
Upper (I)	0	0	0	0	0	10.533	0.015	Yes
Upper middle (II)	4	4.8	6	2.6	10			
Lower middle (III)	1	1.2	28	12.3	29			
Upper lower (IV)	63	75.9	145	63.9	208			
Lower (V)	15	18.1	48	21.1	63			
Total	83	100	227	100	310			

Age

The distribution of glycemic control across the age groups did not indicate a statistically significant association (χ² = 6.179, p = 0.103). Among both the good and poor glycemic control groups, most patients were aged 46-60 years (69.9% and 60.8%, respectively).

Gender

Among the individuals with good glycemic control, 53.0% were female and 47.0% were male. Among those with poor glycemic control, females and males were nearly equally represented (49.8% and 50.2%, respectively).

Anthropometric and metabolic profile

The study analyzed the association between BMI and glycemic control status. Among patients with good glycemic control, 61.4% were obese, 22.9% were overweight, and 15.7% had a normal BMI. In the poor glycemic control group, 55.5% were obese, 21.1% were overweight, and 23.3% had a normal BMI (χ² = 2.147, p = 0.342). No significant association was found between BMI category and glycemic control status (Table 2).

**Table 2:** Anthropometric and metabolic factors affecting glycemic control HDL: high-density lipoprotein; LDL: low-density lipoprotein; TC: total cholesterol; TG: triglycerides; WHR: waist-to-hip ratio

Demographic variables	Good glycemic control		Poor glycemic control		Test	p-Value	Significant at the 5% level
Category of BMI	f	%	f	%	Chi-square test		
Normal	13	15.7	53	23.3	2.147	0.342	No
Overweight	19	22.9	48	21.1			
Obese	51	61.4	126	55.5			
Total	83	100	227	100			
	N	Mean ± SD	N	Mean ± SD	Independent test		
Waist circumference	83	86.44 ± 14.37	227	93.21 ± 12.66	2.148	0.033	Yes
Hip circumference	83	98.33 ± 11.84	227	94.86 ± 10.98	1.278	0.203	No
WHR (female)							
>0.85	28	6.60 ± 0.33	91	8.99 ± 1.55	3.751	0	Yes
≤0.85	18	5.95 ± 0.25	3	9.57 ± 1.97	3.763	0.013	Yes
WHR (male)							
>0.9	0	-	87	9.69 ± 1.60	-	-	-
≤0.9	83	6.38 ± 0.33	46	8.75 ± 1.80	3.690	0.001	Yes
Overall WHR	83	0.88 ± 0.03	227	0.98 ± 0.08	5.264	0	Yes
Lipid profile							
TC	83	180.93 ± 42.40	227	185.01 ± 40.28	0.78	0.436	No
TG	83	158.39 ± 74.98	227	181.53 ± 85.92	2.170	0.031	Yes
HDL	83	51.05 ± 10.06	227	46.78 ± 10.91	3.113	0.002	Yes
LDL	83	108.58 ± 37.83	227	104.71 ± 33.19	0.875	0.382	No
Body fat analysis							
Body fat %	83	19.36 ± 5.40	227	20.34 ± 6.58	0.945	0.346	No
Visceral fat level	83	5.62 ± 2.52	227	5.68 ± 2.71	0.142	0.887	No
Fat mass index	83	4.25 ± 1.41	227	4.51 ± 1.81	0.929	0.354	No
Resting metabolism	83	1267.48 ± 168.94	227	1280.09 ± 215.84	0.374	0.709	No

Significant differences, however, were observed in waist circumference. Patients with poor glycemic control had a higher mean waist circumference (93.21 ± 12.66 cm) compared with those with good glycemic control (86.44 ± 14.37 cm; p = 0.033). No significant difference was noted for hip circumference between the groups (good control, 98.33 ± 11.84 cm; poor control, 94.86 ± 10.98 cm; p = 0.203).

A sex-specific analysis of the WHR showed that among females with WHR > 0.85, those in the poor glycemic control group had significantly higher mean values (8.99 ± 1.55) than those in the good control group (6.60 ± 0.32; p = 0.000). Among females with WHR ≤ 0.85, the poor control group again showed higher mean values (9.57 ± 1.97) than the good control group (5.95 ± 0.25; p = 0.013). Among males with WHR > 0.9, the poor control group had a mean value of 9.69 ± 1.60, while those with WHR ≤ 0.9 had significantly lower values (8.75 ± 1.80) compared with males in the good control group (6.38 ± 0.33; p = 0.001). Overall, WHR was significantly higher in the poor glycemic control group (0.98 ± 0.08) than in the good control group (0.88 ± 0.03; p ≤ 0.001; Table 2).

Lipid Profile

Analysis of lipid parameters revealed statistically significant differences in TG and HDL levels. TG levels were significantly higher in the poor glycemic control group (181.53 ± 85.92 mg/dL) compared with the good control group (158.39 ± 74.98 mg/dL; p = 0.031). Conversely, HDL levels were significantly lower in the poor control group (46.78 ± 10.91 mg/dL) than in the good control group (51.05 ± 10.06 mg/dL; p = 0.002). No statistically significant differences were observed for total cholesterol or LDL levels between the two groups (p > 0.05; Table 2).

Body Fat Analysis

Comparison of body composition parameters between the groups showed no statistically significant differences. The mean body-fat percentage was slightly higher in the poor glycemic control group (20.34 ± 6.58%) than in the good control group (19.36 ± 5.40%; p = 0.346). The mean visceral fat percentage was 5.62 ± 2.52 in the good control group and 5.68 ± 2.71 in the poor control group, with no significant difference (p = 0.887). Patients in the poor control group also had a slightly higher mean fat mass index (4.51 ± 1.81) compared with the good control group (4.25 ± 1.41; p = 0.354). Mean resting metabolism was 1267.48 ± 168.94 kcal/day in the good control group and 1280.09 ± 215.84 kcal/day in the poor control group (p = 0.709; Table 2).

Disease Profile of T2DM Patients

Patients were grouped into three categories according to disease duration: less than five years, five to 10 years, and more than 10 years. Among those with good glycemic control, 43.4% had diabetes for less than five years, 37.3% for five to 10 years, and 19.3% for more than 10 years. Similarly, in the poor glycemic control group, 42.3% had the disease for under five years, 40.1% for five to 10 years, and 17.6% for more than 10 years. The distribution was similar across both groups, and the difference was not statistically significant.

To evaluate the potential influence of age at disease onset on glycemic control, patients were classified into two categories: onset before 50 years of age and onset at or after 50 years of age. Most patients in both groups had disease onset before age 50 (81.9% in the good glycemic control group; 80.6% in the poor control group). The difference was not statistically significant (χ² = 0.068, p = 0.795).

A significant association was observed between family history of diabetes and glycemic control (χ² = 7.858, p = 0.005). Patients with poor glycemic control were more likely to report a positive family history (61.2%) than those with good glycemic control (49.4%), suggesting a possible genetic or familial contribution to poorer outcomes.

The relationship between smoking and glycemic control was also assessed. There was no statistically significant difference in current smoking status between the two groups (χ² = 1.166, p = 0.280). The prevalence of current smokers was low overall: 2.4% in the good glycemic control group and 5.3% in the poor control group. However, among current and former smokers, patients with poor glycemic control reported longer smoking durations, indicating a potential association between cumulative tobacco exposure and impaired glycemic regulation. Among individuals with good glycemic control, the mean smoking duration was four years, notably shorter than that in the poor control group.

A statistically significant association was found between dyslipidemia and glycemic control. Among patients with poor glycemic control, 37.44% had dyslipidemia compared with 21.69% of patients with good control (χ² = 6.803, p = 0.009).

The association between hypertension and glycemic control was also examined. Among patients with good glycemic control, 50.6% had hypertension, compared with 55.95% in the poor control group. This difference was not statistically significant (χ² = 0.700, p = 0.403; Table 3).

**Table 3:** Disease-related factors affecting glycemic control DBP: diastolic blood pressure; SBP: systolic blood pressure

Variable	Good glycemic control		Poor glycemic control		Total	Chi-square test	p-Value	Significant at the 5% level
	f	%	f	%				
Duration category (years)								
≤5	36	43.4	96	42.29	132	0.225	0.894	No
5-10	31	37.3	91	40.1	122			
>10	16	19.3	40	17.6	56			
Age of onset category								
≤50	68	81.9	183	80.6	251	0.068	0.795	No
50 and above	15	18.1	44	19.4	59			
Number of complications								
Yes	7	8.43	14	6.17	21	0.494	0.482	No
No	76	91.57	213	93.83	289			
Family history of diabetes								
No	47	50.6	88	38.8	135	7.886	0.005	Yes
Yes	36	49.4	139	61.2	175			
Currently smoking								
No	81	97.6	215	94.7	296	1.166	0.280	No
Yes	2	2.4	12	5.3	14			
Duration of smoking (years)								
3	0	0	3	25	3	5.833	0.212	No
4	2	100	2	16.7	4			
5	0	0	4	33.3	4			
6	0	0	1	8.3	1			
10	0	0	2	16.7	2			
Total	2	100	12	100	14			
Presence of dyslipidemia								
Yes	18	21.69	85	37.44	103	6.803	0.009	Yes
No	65	78.31	142	62.56	207			
Presence of hypertension								
Yes (SBP >140, DBP >90)	42	50.6	127	55.95	169	0.700	0.403	No
No	41	49.4	100	44.05	141			

Therapy-related factors affecting glycemic control

A statistically significant association was found between insulin use and glycemic control (χ² = 4.034, p = 0.045). Among patients with good glycemic control, 8.43% were on insulin therapy, compared with 3.08% of those with poor glycemic control. The higher proportion of insulin users in the good glycemic control group may reflect a more intensively managed subgroup or variability in treatment responsiveness (Table 4).

**Table 4:** Antidiabetic therapy-related factors affecting glycemic control DPP-4: dipeptidyl peptidase-4; SGLT2: sodium/glucose cotransporter 2

Drug	Good glycemic control		Poor glycemic control		Total	Chi-square test	p-Value	Significant at the 5% level
	f	%	f	%				
Insulin								
No	76	91.57	220	96.92	296	4.034	0.045	Yes
Yes	7	8.43	7	3.08	14			
Number of drugs								
1	8	9.64	3	1.32	11	19.822	0.001	Yes
2	20	24.1	51	22.47	71			
3	20	24.1	77	33.92	97			
4	15	18.07	49	21.59	64			
5	20	24.1	37	16.3	57			
6	0	0	10	4.41	10			
Biguanide								
No	1	1.2	0	0	1	2.744	0.098	No
Yes	82	98.8	227	100	309			
Sulfonylureas								
No	32	38.55	76	33.48	108	0.689	0.406	No
Yes	51	61.45	151	66.52	202			
DPP-4 inhibitors								
No	33	39.76	63	27.75	96	4.098	0.043	Yes
Yes	50	60.24	164	72.25	214			
α-Glucosidase inhibitors								
No	56	67.47	138	60.79	194	1.157	0.282	No
Yes	27	32.53	89	39.21	116			
SGLT2 inhibitor								
No	51	61.45	122	53.74	173	1.462	0.227	No
Yes	32	38.55	105	46.26	137			
Thiazolidinediones								
No	67	80.72	211	92.95	278	9.918	0.002	Yes
Yes	16	19.28	16	7.05	32			
Meglitinide/phenylalanine analogues								
No	79	95.18	224	98.68	303	3.369	0.066	No
Yes	4	4.82	3	1.32	7			

A significant association was also observed between the number of antidiabetic drugs prescribed and glycemic control status (χ² = 19.822, p = 0.001). In the good glycemic control group, patients were fairly evenly distributed across the use of two to five medications: 24.1% were taking either two, three, or five drugs, while 18.07% reported using four drugs. Only 9.64% were on monotherapy (one drug), and no patients were on a six-drug regimen.

In contrast, among patients with poor glycemic control, the largest proportion (33.92%) was receiving three-drug therapy, followed by 22.47% on a two-drug regimen and 21.59% on a four-drug regimen. A further 16.30% were taking five medications, and 4.41% were on six drugs, suggesting potentially severe disease or pharmacological resistance. This statistically significant pattern indicates that poor glycemic control is associated with the need for more complex and intensive pharmacological management, possibly reflecting treatment resistance or more advanced disease.

The use of biguanides was widespread in both groups, with 98.8% of patients with good glycemic control and 100% of those with poor control receiving this medication (χ² = 2.744, p = 0.098). There was no significant association between biguanide use and glycemic control. Similarly, sulfonylurea use did not differ significantly: 61.45% of patients with good glycemic control and 66.52% with poor control were on these drugs (p = 0.406).

In contrast, a significant difference was observed in the use of DPP-4 inhibitors. Among patients with poor glycemic control, 72.25% were taking DPP-4 inhibitors, compared with 60.24% in the good glycemic control group (χ² = 4.098, p = 0.043). By comparison, α-glucosidase inhibitor use showed no statistical association with glycemic control (χ² = 1.157, p = 0.282). In the good glycemic control group, 67.47% of patients were not taking α-glucosidase inhibitors, whereas 32.53% were. In the poor control group, 60.79% were nonusers and 39.21% were users.

Use of sodium/glucose cotransporter 2 (SGLT2) inhibitors also showed no significant association (χ² = 1.462, p = 0.227). In the good glycemic control group, 38.55% of patients were users and 61.45% were nonusers. Among those with poor control, 46.26% were on SGLT2 inhibitors and 53.74% were not. Although use was slightly higher in the poor control group, the difference was not statistically significant.

In contrast, thiazolidinedione use showed a highly significant association with glycemic control (χ² = 9.918, p = 0.002), suggesting a potentially meaningful impact of this drug class on outcomes. Only 7.05% of patients in the poor control group were on thiazolidinediones, compared with 19.28% in the good control group.

The use of meglitinides and phenylalanine analogues showed a trend toward significance (χ² = 3.369, p = 0.066). In the good glycemic control group, 95.18% were not taking this therapy and 4.82% were, while in the poor control group, 98.68% were nonusers and 1.32% were users (Table 4).

Diabetes self-care practices

**Figure 1:**
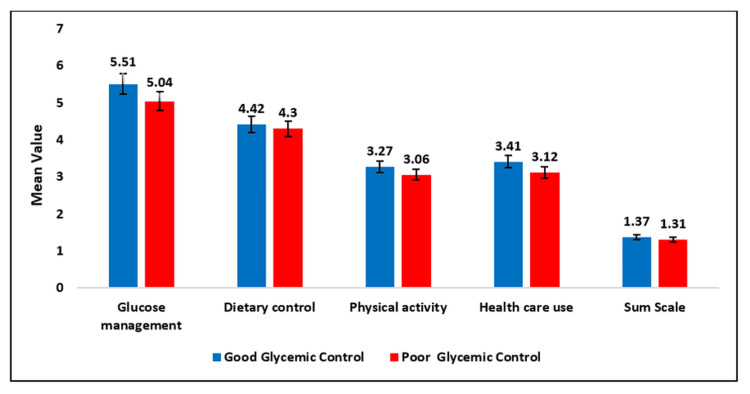
Self-care practices among diabetic patients

Figure 1 presents a comparison of glycemic control status with various self-care parameters among patients with T2DM. Glucose management scores were significantly higher in the good glycemic control group (5.51 ± 1.22) than in the poor control group (5.04 ± 1.31; t = 2.803, p = 0.005). Physical activity also showed a significant association with glycemic control, with higher scores observed in the good control group (3.27 ± 0.74) compared with the poor control group (3.06 ± 0.75; t = 2.179, p = 0.030). Health care use was likewise significantly greater in the good glycemic control group (3.41 ± 1.50) than in the poor control group (3.12 ± 0.91; p = 0.043), indicating a meaningful impact of healthcare utilization on glycemic control.

In contrast, dietary control and the composite sum scale did not show significant differences between the two glycemic control groups (p = 0.452 and p = 0.442, respectively). The total behavioral score was slightly higher in the good glycemic control group (17.71 ± 3.11) compared with the poor control group (17.10 ± 2.67), but this difference was not statistically significant (t = 1.714, p = 0.088). These findings underscore the role of glucose monitoring, physical activity, and healthcare utilization as key behavioral determinants associated with better glycemic control in patients with T2DM (Figure 1).

**Figure 2:**
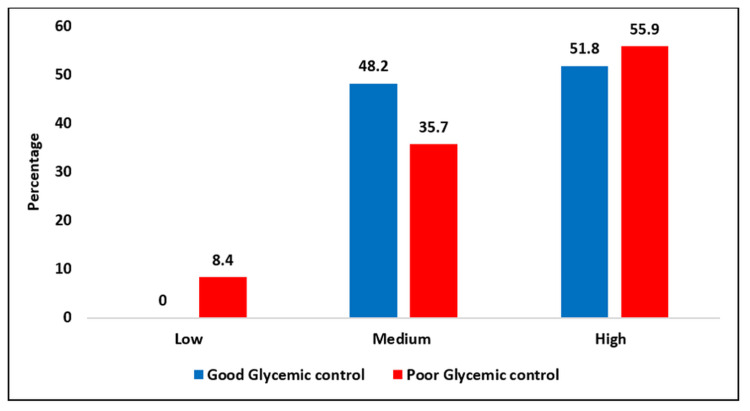
Medication adherence patterns among diabetic patients The MMAS-4 Scale, content, name, and trademarks are protected by US copyright and trademark laws. Permission for use of the scale and its coding is required. A license agreement is available from MMAR, LLC., www.moriskyscale.com.

Medication adherence patterns were assessed using the MMAS-4. Figure 2 shows the association between glycemic control and medication adherence patterns based on the chi-square test. A statistically significant relationship was found between adherence and glycemic control (χ² = 9.574, p = 0.008), indicating that better adherence was associated with better glycemic control. Among patients with good glycemic control, 51.8% demonstrated high adherence and 48.2% medium adherence, while none had low adherence. In contrast, among patients with poor glycemic control, 8.4% showed low adherence, 35.7% medium adherence, and 55.9% high adherence. Notably, low adherence was observed exclusively in the poor control group (Figure 2).

Diabetes distress

The analysis of diabetes distress in relation to glycemic control revealed a significant association (χ² = 9.6824, p = 0.007). Among patients with good glycemic control, 42% reported no distress, compared with only 17% in the poor control group. High distress levels were more frequent in the poor control group (25%) than in the good control group (8%). Moderate distress was the most common category in both groups, reported in 49% of patients with good control and 58% of those with poor control. These findings highlight the impact of psychological distress on diabetes management and emphasize the need to incorporate distress management into routine care to support optimal glycemic control.

## Discussion

T2DM is a chronic metabolic disorder that has reached epidemic proportions globally. Despite advancements in pharmacological therapies and healthcare infrastructure, maintaining optimal glycemic control remains elusive for many patients. Poor glycemic control is a key contributor to long-term complications such as nephropathy, retinopathy, and cardiovascular disease [16]. This study assessed the demographic, anthropometric, metabolic, therapeutic, and psychological determinants of glycemic control in patients with T2DM. The objective was to identify barriers and predictors of poor glycemic control, with a view to guiding targeted interventions for improved outcomes.

Prevalence and burden of glycemic dysregulation

The results showed that 73% of patients had poor glycemic control, a finding consistent with similar studies conducted in India and globally. A series of multicenter investigations across India have reported suboptimal glycemic control among people with T2DM. The A1chieve study, encompassing over 20,000 patients, documented a mean HbA1c of 9.2%, with delayed insulin initiation and clinical inertia identified as key contributors [17]. The ICMR-INDIAB study similarly found that only 30% of known diabetics achieved their glycemic targets, with disparities across urban and rural populations [17]. National data from the TIGHT study showed that 76.6% of patients had HbA1c levels above 7%, with poorer control linked to obesity, hypertension, and longer disease duration.

Region-specific findings confirm these trends. For example, a South Indian tertiary center reported unsatisfactory fasting blood sugar levels in 58% of patients; similarly, a North Indian facility noted poor glycemic control in 57.6%, attributed to sociodemographic and behavioral factors. Collectively, these studies underscore the need for earlier intervention, tailored education, and enhanced adherence strategies to improve diabetes outcomes across diverse Indian populations [18].

Sociodemographic determinants of glycemic control

Although the association did not reach statistical significance, younger individuals (18-35 years) tended to exhibit poorer glycemic control than older patients. This finding may reflect delayed disease recognition and less structured self-management among young patients. Similarly, data from NFHS-5 (2019-2021) indicate that more than half of diabetic patients aged 15-49 years who received treatment had poorly controlled blood glucose levels, with a notable burden among the youngest adults [19].

Gender-related differences were minimal, consistent with findings from Malaysian and South African cohorts, as well as Indian tertiary care data [20]. These results indicate a lack of sex-based disparity. However, a statistically significant association (p = 0.015) was observed between lower socioeconomic status and poor glycemic control. Similar findings were reported by Mohan et al., who showed that income and education levels strongly influenced medication adherence and dietary practices [20].

Anthropometric and Metabolic Factors

BMI scores were not significantly associated with glycemic control. However, more than 60% of patients with poor glycemic control were overweight or obese. The Look AHEAD trial (2013) established that weight reduction significantly improves insulin sensitivity and glycemic parameters [21]. In the present study, elevated TG (p = 0.031) and reduced HDL levels (p = 0.002) were significantly associated with poor glycemic control. These results align with the Framingham Heart Study, which correlated dyslipidemia with insulin resistance and poor glycemic trajectories [22].

Anthropometric assessments also indicated the relevance of central obesity indicators, such as WHR, along with conventional metrics such as BMI and visceral fat. Elevated WHR, a surrogate marker for abdominal adiposity, is increasingly recognized as a strong predictor of insulin resistance and poor glycemic control. Research from Madhya Pradesh identified a negative association between WHR and HDL cholesterol in prediabetic patients, implicating WHR in early cardiometabolic risk profiling [16]. Similarly, the PERSIAN Guilan cohort reported elevated WHR in association with age, female sex, and increased fasting blood glucose, suggesting the predictive utility of this measure across demographic categories. Comparative data from Ethiopia demonstrated that WHR and the waist-to-height ratio outperformed BMI for identifying poor glycemic control, with WHR achieving an area under the curve of 0.64 [23]. These findings affirm WHR as a practical and informative metric for metabolic risk stratification, supporting its incorporation into diabetes screening and personalized care strategies [16,23].

Analysis of BMI and body composition parameters revealed a metabolically adverse profile indicative of overweight and adiposity-driven insulin resistance. Visceral fat levels (mean: 5.67 ± 2.66) and fat mass index scores (4.46 ± 1.73) further highlight the contribution of central obesity to impaired glycemic regulation, as reflected in a mean HbA1c of 9.05 ± 2.39%. These findings are consistent with the A1chieve study, which reported a mean HbA1c of 9.2% among Indian T2DM patients, linking poor glycemic control to delayed insulin initiation and excess adiposity [20].

A pedometer-based intervention trial by Rekha et al. demonstrated that structured physical activity significantly reduced HbA1c and body fat percentages over 12 weeks, highlighting the role of lifestyle modification in improving metabolic outcomes [24].

Disease-Specific Parameters

The duration of diabetes and age at onset showed no significant statistical relationship in this cohort. However, longer disease duration is widely recognized as increasing the risk of poor glycemic control. A positive association (p = 0.005) was observed between family history of diabetes and poor glycemic control, consistent with findings from genetic epidemiology, which suggest hereditary predisposition may alter beta-cell reserves and accelerate glucose dysregulation. Dyslipidemia was also significantly linked to poor glycemic control (p = 0.009). Hypertension, however, was not associated with poor glycemic control, although other studies, such as the ADVANCE trial, have shown that blood pressure control is crucial for reducing microvascular complications [25].

Therapy-Related Dynamics

Patients receiving insulin therapy exhibited statistically poorer glycemic control (p = 0.045). This finding may reflect delayed insulin initiation or progression to an advanced stage of diabetes. The burden of managing multiple medications was also notable; patients on multiple drugs showed poorer glycemic control than those with good control (p = 0.001). This finding is consistent with the DAWN2 study, which highlighted polypharmacy as a barrier to adherence and treatment satisfaction [26].

The use of DPP-4 inhibitors (p = 0.043) and thiazolidinediones (p = 0.002) was associated with suboptimal glycemic outcomes, despite their proven pharmacological efficacy. These results suggest that real-world effectiveness may depend on dose adjustments, timely therapeutic escalation, and patient-specific factors such as compliance, tolerability, and disease severity [27].

Psychological and Behavioral Considerations

The findings highlight the influence of specific self-care behaviors on glycemic regulation. Marked differences in glucose monitoring, physical activity, and healthcare engagement between patients with well-controlled and poorly controlled diabetes indicate that consistent self-monitoring, regular exercise, and proactive healthcare utilization are essential for achieving stable glycemic outcomes.

In contrast, the absence of significant variation in dietary control may reflect challenges such as cultural influences, reporting inconsistencies, or overlapping effects with other behavioral domains. This warrants further exploration into contextual determinants of dietary adherence. Disaggregated analysis of self-care dimensions provides valuable insight into patient-specific behaviors, enabling the development of tailored educational models and predictive tools for Indian diabetic populations [28].

The study also established a statistically significant link between diabetes distress and glycemic control (χ² = 9.6824, p = 0.007). Among patients with good glycemic control, 42% reported no distress compared with only 17% in the poor control group. High distress levels were more frequent in the poor control group (25%) than in the good control group (8%). Moderate distress was the most common category in both groups (49% and 58%, respectively). These results underscore the impact of psychological distress on diabetes management and highlight the need for integrating distress management into routine care [29].

The significant association between medication adherence and glycemic control (χ² = 9.574, p = 0.008) further highlights the critical role of self-management in diabetes care. No cases of low adherence were reported among patients with good glycemic control, whereas low adherence occurred only in the poor control group. High adherence was strongly associated with better glycemic outcomes, consistent with findings by Lin et al., who linked poor adherence to elevated HbA1c levels in newly diagnosed patients [30]. These insights validate medication adherence as a strong indicator of metabolic stability and confirm the need for individualized support strategies that address both pharmacological and nonpharmacological barriers to optimal diabetes management.

Future research should explore how changes in emotional distress, self-care, and adherence affect glycemic control over time. Culturally adapted interventions, such as peer support and digital tools, may help address these behavioral factors. Routine screening for distress and adherence in clinical settings could enhance personalized care, particularly in urban, resource-limited contexts. Incorporating these insights into predictive models and medical education has the potential to improve diabetes management and training.

Limitations

This study has several limitations. First, the modest sample size may not accurately reflect the larger population, limiting generalizability. Second, the cross-sectional design precludes conclusions about causal relationships. Third, reliance on self-reported data introduces potential biases, including recall errors and socially desirable responses. Finally, the study’s focus on a specific geographic and cultural population may limit the applicability of the findings to other contexts.

## Conclusions

This study highlights the multifaceted nature of glycemic regulation, showing that self-care adherence, emotional distress, and socioeconomic context play pivotal roles in effective diabetes management. The strong associations observed between adherence and glycemic control underscore the importance of routine monitoring, medication compliance, and regular healthcare engagement in achieving metabolic stability. The prevalence of diabetes-related distress, even among individuals with good glycemic outcomes, reveals the psychological toll of persistent self-care. Such distress may act both as a barrier and as a motivator in glycemic management. Collectively, the findings suggest the need for a personalized, context-sensitive approach to diabetes care that integrates behavioral and psychosocial support strategies tailored to the Indian diabetic population.
